# Same-Day Discharge After Left Atrial Appendage Closure: Ready for Prime Time but for Whom?

**DOI:** 10.1016/j.shj.2026.101074

**Published:** 2026-06-24

**Authors:** Pedro Cepas-Guillén, Maria Teresa Espinosa Moreno, Eduardo Flores-Umanzor, Laura Sanchis, Xavier Freixa

**Affiliations:** Department of Cardiology, Institut Cardiovascular, IDIBAPS, Hospital Clinic Barcelona, Barcelona, Spain

Transcatheter left atrial appendage closure (LAAC) is gaining interest as an alternative strategy for stroke prevention in selected patients with atrial fibrillation who are not candidates for long-term oral anticoagulation.^1^ As experience with the technique has increased, attention has progressively shifted from procedural feasibility to the optimization of procedural care. In this context, same-day discharge (SDD) after LAAC represents an attractive strategy, with potential benefits for patients and hospital resource utilization.

In the present study, Hyun et al.^2^ provide an analysis of SDD after LAAC using the Nationwide Readmissions Database from 2016 to 2020. Including 40,564 index LAAC hospitalizations with a length of stay of 0 or 1 day, the authors compared patients discharged on the same day with those kept overnight. Overall, SDD remained uncommon, accounting for only 4.1% of the cohort but increasing over time from 1.6% in 2016 to 7.7% in 2020. Importantly, SDD was not associated with higher early readmission either at 7 days or at 30 days. Crude 7-day readmission was 2.0% after SDD versus 2.4% after overnight stay, whereas 30-day readmission was 7.0% versus 7.6%, respectively. After multivariable adjustment, SDD was not associated with increased 7-day or 30-day readmission.

The authors should be congratulated for conducting the largest analysis of this topic in the LAAC field. These findings are clinically relevant for several reasons. First, the authors addressed a common clinical question: whether a patient with an uncomplicated LAAC procedure requires routine overnight observation or can be safely discharged on the same day. By excluding patients who stayed in the hospital for 2 or more days, the authors reduced a potential bias when comparing SDD with patients whose longer hospitalization may have been related to complications, frailty, slower recovery, or other clinical issues.

Second, the inclusion of 7-day readmission as an endpoint is particularly important. The absence of an increased 7-day readmission signal provides reassurance that, in selected patients, SDD does not appear to shift early postprocedural complications from the hospital to the outpatient setting. Prior studies have focused primarily on the 30-day readmission endpoint,^3^ which may capture events that are less directly related to the immediate discharge decision, whereas very early readmission better reflects the safety of postprocedural monitoring.^4^

Third, the results are aligned with previous studies evaluating SDD after LAAC.^3^ The available evidence in this field suggests that SDD can be performed safely in selected patients without an apparent increase in adverse events or early readmission compared with overnight observation. The present study reinforces these observations in a larger national cohort and adds further support to the feasibility of SDD as part of contemporary LAAC practice.

Nevertheless, the results should be interpreted with caution. The most important limitation is selection bias. Patients discharged on the same day were probably those with uncomplicated procedures, favorable recovery, and adequate social support. These variables are not fully captured in administrative databases. Therefore, the study should not be interpreted as evidence that SDD is appropriate for all patients undergoing LAAC. Rather, it supports the safety of SDD in carefully selected patients after an uncomplicated procedure.

The study also lacks important procedural details, including device type, anesthesia and imaging strategy, vascular access management, antithrombotic therapy, and, most importantly, the specific discharge criteria used. These factors are central to clinical decision-making after LAAC. In addition, the dataset includes procedures only through 2020, and contemporary practice may differ substantially due to newer-generation devices, increasing procedural experience, refined imaging protocols, and more established structural heart pathways. Therefore, the absence of granular data regarding procedural care pathways remains one of the main limitations of studies based on large administrative registries.

In our experience, SDD after LAAC should be viewed not as a “shortcut” to reduce length of stay, but as the final step of a structured care pathway. Such a pathway should include careful patient selection, procedural simplification through light sedation, and the use of miniaturized transesophageal echocardiography guidance^5^^,^^6^; confirmation of procedural success; exclusion of relevant periprocedural complications, especially pericardial effusion and vascular complications; clear antithrombotic instructions; patient education, including recognition of complications and appropriate actions if they occur; and early postdischarge follow-up (Figure 1). Importantly, this pathway requires close coordination, with an advanced practice nurse playing a central role in patient education, discharge suitability assessment, and early postdischarge contact. Following these principles, SDD is being increasingly adopted across percutaneous cardiovascular interventions, including complex structural procedures such as transcatheter aortic valve implantation.^7^

**Figure 1:**
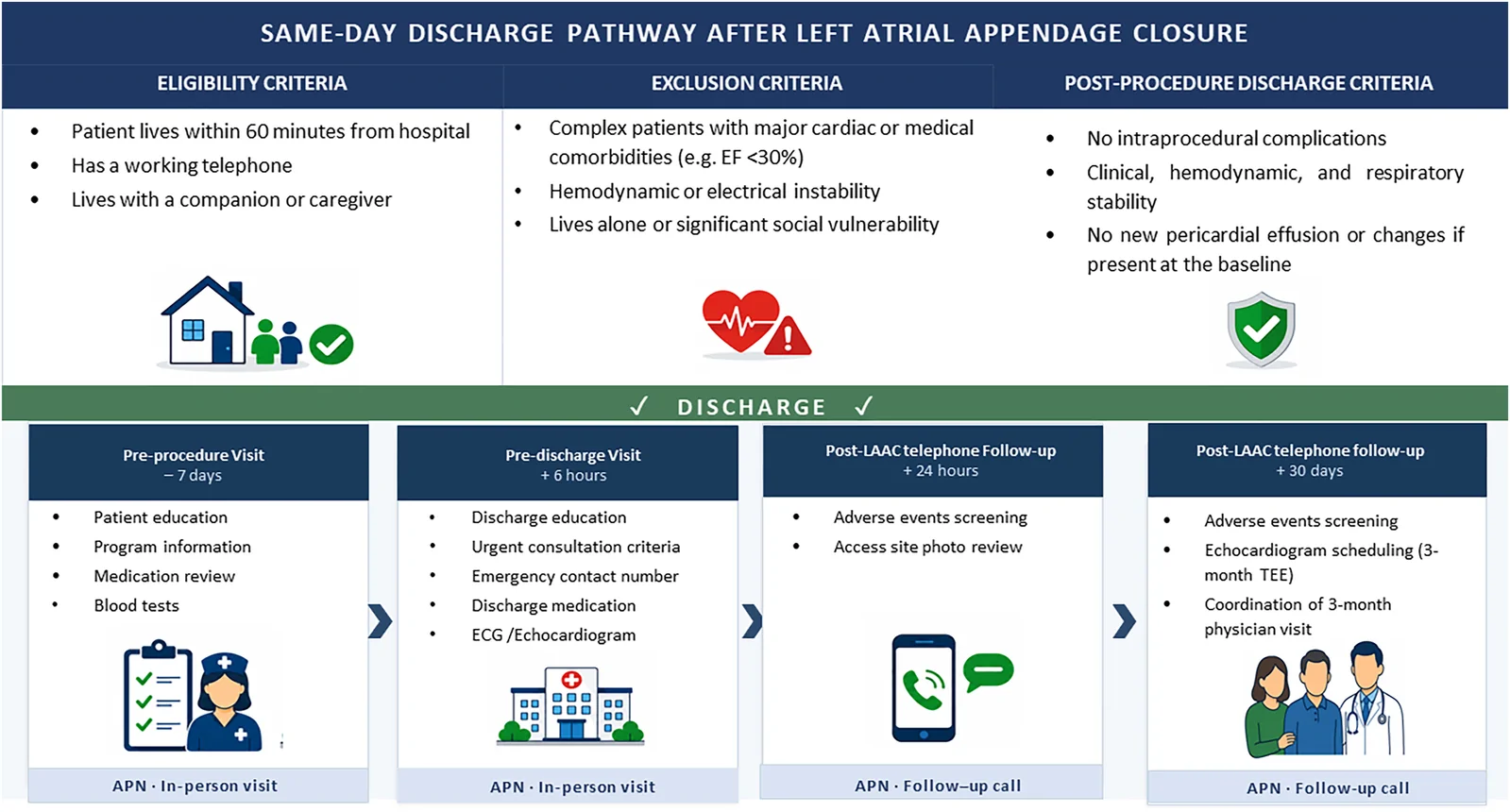
**Same-day discharge pathway after percutaneous left atrial appendage closure.** The pathway includes patient selection, postprocedural discharge criteria, discharge education, and structured early follow-up. Abbreviations: APN, advanced practice nurse; ECG, electrocardiogram; EF, ejection fraction; TEE, transesophageal echocardiogram.

In conclusion, the study by Hyun et al. provides important evidence supporting the feasibility of selective SDD after LAAC. As the use of LAAC continues to increase, the next step is not simply to determine whether SDD can be performed safely, but to identify which patients are suitable candidates and what measures are needed to ensure safe implementation. SDD after LAAC appears to be a reasonable option in selected patients, provided that it is supported by structured protocols and careful clinical assessment.

## Funding

The authors have no funding to report.

## Disclosure Statement

Laura Sanchis and Xavier Freixa are proctors for Abbott Medical. The other authors had no conflicts to declare.
